# Effect of Urbanization on the River Network Structure in Zhengzhou City, China

**DOI:** 10.3390/ijerph19042464

**Published:** 2022-02-21

**Authors:** Hongxiang Wang, Lintong Huang, Jianwen Hu, Huan Yang, Wenxian Guo

**Affiliations:** School of Water Conservancy, North China University of Water Resources and Electric Power, Zhengzhou 450045, China; whxzju@163.com (H.W.); 201604630@stu.ncwu.edu.cn (L.H.); 201602623@stu.ncwu.edu.cn (J.H.); yanghuan202201@163.com (H.Y.)

**Keywords:** urbanization, river network structures, modified normalized difference water index, entropy weight method

## Abstract

Hydrological problems, such as flood disasters, can be caused by the influence of urbanization on river network structures in plain areas. Taking the main urban region of Zhengzhou city as the research area, based on six remote sensing images from 1992 to 2015, the modified normalized difference water index method and a land-use transfer matrix were used to reconstruct river network data to study the temporal and spatial changes in the river system. In addition, the analytic hierarchy process and the entropy weight method were used to construct pattern indexes of the river system to quantitatively evaluate the inner relationship between the urbanization process and the river network structure in the plain area. The results showed that the percentages of arable land, forest and grassland, water, and unused land in Zhengzhou that was transferred to construction land from 1992 to 2015 were 59.10%, 51.05%, 29.83%, and 58.76%, respectively. In the past 34 years, the morphological indices, structural indices, and connectivity indices of the river system experienced a trend of high to low, and then increased, with the structural indices being significantly correlated with construction land use (*p* < 0.05). The regression equation R^2^ between urbanization level and river length, water area, river network density, water surface rate, connection rate, and connectivity ranged from 0.677 to 0.966, which could well reflect the response relationship between urbanization and the river network. In addition, the outflow was greater than the inflow, which has destroyed the natural structure of the channel.

## 1. Introduction

River networks are important for the survival and reproduction of animals and plants, supporting various functions of human society and essentially maintaining ecological stability [1,2]. Since the 20th century and during the period of rapid urbanization, human activities associated with urbanization have resulted in nearly 60% of the changes in rivers worldwide [3,4]. Typical rivers in northern China have been affected by natural changes and human activities during their evolution, and the growth of these rivers has been strongly impacted by human activities over recent years. Over short time scales, human activities determine changes in the structural details of river networks. The structural characteristics of river networks have an important influence on their storage function, and a reduction in the storage capacity of river networks due to urbanization development is the main cause of current urban flooding [5]. In general, ecological problems are becoming increasingly prominent, a fact which seriously threatens the sustainable development of human society [6].

River networks have a variety of ecosystem services [7]. Neeson (2011) reported on the effects of a river network structure on adult lampreys and their larvae [8]. Knowing the structure of a river network can also help to identify the location of wastewater discharge and reduce the impact of microbial contamination in the river [9]. The impact of urbanization on the structure of river networks has long been a concern [10]. Many highly urbanized areas have lost at least 85% of their river channels, and metropolises such as Chicago have seen the phenomenon of urban stream deserts [11]. The coral reef is an important ecosystem. It not only provides shelter and breeding grounds for many marine species, but it also controls the level of carbon dioxide and affects the development of urban ecosystems [12]. The water system structure and environmental function of a river basin can only serve humans for a long time if it is intact. Therefore, some scholars have integrated multiple factors to establish indicators to evaluate river basin systems. For example, Grill (2014) introduced the River Connectivity Index and the River Regulation Index to evaluate the cumulative impact of dams on the Mekong River Basin [13]. Saunders (2016) used graph theory to quantify the connectivity of marine ecosystems, helping to determine the generality of human impact [14]. The river ecosystem is complex, and it is difficult to evaluate its health status by establishing a comprehensive index system. Therefore, an effective way to solve this problem is to construct an index evaluation system that is suitable for local river systems based on the actual situation of the target. 

Among the regional characteristics of the landscape, the spatial structure of the water system features metric analysis indicators that mainly include the length of the river, node connection rate, river network density, etc. The length is used to determine the degree of contact between the river and the matrix. Node connection rate refers to the degree of spatial continuity of the channel, and whether the channel is disconnected is an important factor in determining its barrier function benefits. Therefore, node connection rate is an important metric of river structure. River network density is used to describe the density of river channels. Phillips (2011) quantified the dynamic hydrological connectivity in heterogeneous basins using a hydrological connectivity function based on graph theory [15]. Meng et al. (2014) believe that the reduced connectivity of river system structures is closely related to the frequent occurrence of flood disasters in modern cities [16]. Lin et al. (2019) used Suzhou city as an example to discuss the relationship between changes in the plain river system and its hydrological characteristics under the influence of urbanization [17]. However, there is insufficient research on the extent to which the river system in the typical northern plain cities is affected by urbanization.

Zhengzhou is one of the most typical cities with regard to the development of urbanization in the Central Plains, and it is also a typical representative of the plain river network in northern China. There are many rivers and lakes in the main urban area of Zhengzhou city, with a crisscross water system and a large water surface ratio. Since the 1990s, the economy of the main urban area has developed rapidly. At the same time, with a series of development plans, such as transportation and housing, the regional inland river network has shrunk, and the impervious area has significantly increased. The relationship between urbanization and river systems has become an important issue to be solved urgently. 

Therefore, the objectives of this study were to analyze the temporal and spatial variation characteristics of the river network structure and water system pattern, and quantitatively describe the evolutionary relationship of the regional population, economy, landscape, and urban facilities with the river network in northern China by setting urbanization indicators. The findings of this study could provide a good reference for the protection and development of regional river network structures and water systems in the process of urbanization.

## 2. Materials and Methods

### 2.1. Study Area and Dataset

**Figure 1:**
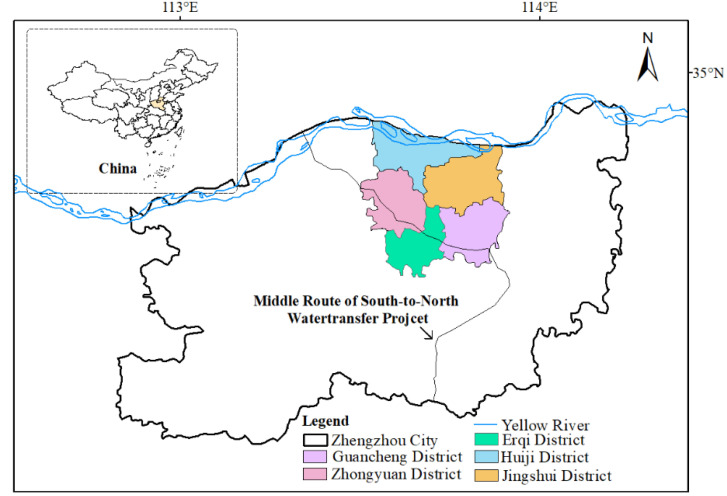
Location of Zhengzhou city.

The surface water system of Zhengzhou city (34°16′–34°58′ N, 112°42′–114°13′ E) belongs to the Yellow River and Huaihe River systems (Figure 1). Zhengzhou is the capital of Henan Province, China, and is located in the north of the central part of Henan Province. The city is 135–143 km long from east to west, 70–78 km wide from north to south, with a total area of 7446 km^2^. We chose urban centers consisting of five major economic zones in Zhengzhou as the representative study area. From 1992 to 2015, the five major economic zones enjoyed rapid economic growth and high urbanization levels, thus showing excellent representativeness. The area of the study is 1010 km^2^, and the terrain is mainly plain. The city’s lakes and reservoirs cover an area of 791.3 km^2^. There are more than 120 rivers, with 29 of those having large drainage areas. The transit rivers are mainly the Yellow River and the Yiluo River. For many years, the average total transit flow reached 44.41 billion m^3^. Zhengzhou is located at the junction of the middle and lower reaches of the Yellow River, and the north-eastern wing of the Funiu Mountains to the Huanghuai Plain. It is high in the west, low in the east, high in the middle, and low in the northeast and southeast. Recently, the rapid urbanization process in Zhengzhou has profoundly and drastically changed the development of the river system in the transition zone of the Huanghuai Plain. 

In view of the typicality of the river network in Zhengzhou city, the characteristics of the underlying surface and the river network in the main urban area of Zhengzhou city were studied using remote sensing technology. The remote sensing data was selected from the TM (thematic mapper) images of the landsat5 satellite in 1992, 2002, 2007, 2010, 2013, and 2015. The original data is from the *China Urban Construction Statistical Yearbook* (1992–2015), the *China Urban Statistical Yearbook* (1992–2015), and the *Zhengzhou Statistical Yearbook* (1992–2015).

### 2.2. Methods

#### 2.2.1. Urbanization Level Assessment

Urbanization level is an important standard to measure the development of regional urbanization, and can reflect the level of socio-economic development of an area more intuitively and accurately. Therefore, this study uses the analytical hierarchy process (AHP) method to quantitatively evaluate the level of urbanization in the region by constructing levels. AHP is superior for solving complex multi-criteria and multi-objective problems. It is a method that first determines the inner connection and hierarchy among indicators by qualitative analysis, and then calculates the weights of related indicators using quantitative analysis and constructing matrices with the criteria [18]. 

We took the level of urbanization development as the target layer, and the four aspects of population, economy, regional landscape, and urban facilities as the criterion layer. The indicator layer includes 10 indicators, such as the total urban population and population density. The comprehensive evaluation index system is shown in Table 1.

In the table, the level of urbanized population is mainly reflected in the two aspects of population size and density. The selected indicators include the total urban population and the density of the urban population. The indicators selected for evaluating the level of economic urbanization are the GDP and the proportion of tertiary industry in GDP. The indicators selected to evaluate the urbanization level of the regional landscape are the amount of built-up area and the area of public green space per capita. The indicators for evaluating the urbanization level of the infrastructure are the length of the water supply pipe, the length of the road, and the area of the road.

The indicators in the quantitative urbanization level index system selected in this paper have different dimensions and orders of magnitude. Therefore, the extreme difference standardization method is first used to unify the data, and then the entropy weight method is used to obtain the composite urbanization index [19]. The reference formula is as follows. 

(1) Generating the Base Matrices

The actual values corresponding to each indicator at different selection points are formed into a base matrix:

(1) $$Z={\left(z_{ij}\right)}_{m\times n}$$

where $z_{ij}$ represents the original values of the *j*th indicator at the *i*th selection point, and *m* and *n* are the number of rows and columns of the matrix, respectively.

(2) Data Standardization Processing Method

When the indicator is positive:

(2) $$X_{ij}=\frac{Z_{ij}-{Z_{ij}}_{min}}{{Z_{j}}_{max}-{Z_{j}}_{min}}$$

When the indicator is negative:

(3) $$X_{ij}=\frac{{Z_{ij}}_{max}\;-Z_{ij}}{{Z_{j}}_{max}-{Z_{j}}_{min}}$$

As shown in Equations (2) and (3), $\;X_{ij}$ is the normalized data, reflecting the size of the information provided by indicator *j*, with the higher values indicating that the indicator is more useful; and ${Z_{ij}}_{max}$ and ${Z_{ij}}_{min}$ are the maximum and minimum values of the original values, respectively. 

(3) Determination of the Index Weights-Entropy Method

Index weight calculation:

(4) $$e_{j}=-k\sum_{i=1}^{m}p(X_{ij})\ln\;p(X_{ij})$$

in the formula, $p_{ij}=\frac{X_{ij}}{\sum_{i=1}^{m}X_{ij}},\;k>0$, to calculate the entropy value of the *j*th index:

(5) $$e_{j}=-k\sum_{i=1}^{m}p_{ij}\ln\;p_{ij}$$

where k>0, ln* is the natural logarithm, $e_{j}\geq 0$. If all j for the $X_{ij}$ given are equal, then $e_{j}=-k\sum_{i=1}^{m}\frac{1}{m}\ln\frac{1}{m}=k\ln m$. If we set $k=\frac{1}{\ln m}$, then $0\leq e_{j}\leq 1$.

To calculate the coefficient of difference for index *j*:

(6) $$g_{j}=1-e_{j}$$

where $g_{j}$ is the coefficient of difference. The higher the value of the coefficient of difference, the greater the amount of information, and the greater the weight value [20].

To calculate the weight:

(7) $$a_{j}:a_{j}=g_{j}/\sum_{j=1}^{n}g_{j}$$

To calculate the comprehensive score:

(8) $$v_{i}=\sum_{j=1}^{n}a_{j}p_{ij}$$

#### 2.2.2. Water System Pattern

Water System Extraction

ENVI (Environment for Visualizing Images) is a complete platform for remote sensing image processing. In this paper, ENVI was used to preprocess the remote sensing image of Zhengzhou and analyze the land-use types. First, the remote sensing scene is classified using the neural network classification method, which is often used for non-linear classification, and is a type of supervised classification [21].

The multi-band method is a method of extracting a water body by analyzing the differences between the spectral curves of the water body and the background features, analyzing the combined advantages of different bands, and establishing a logical expression. McFeeters (1996) proposed the Normalized Difference Water Index (NDWI) to delineate open water features [22]. This is a water body index method proposed on the basis of the multi-band method. This index maximizes the reflectance of water using green light wavelengths, and minimizes the low reflectance of the near-infrared band caused by water features while taking advantage of the high reflectance of the near-infrared band caused by vegetation and soil features. As a result, water features are enhanced owing to having positive values, and vegetation and soil are suppressed due to having zero or negative values. However, the extracted water information in some regions is often mixed with built-up land noise because many built-up areas also have positive values in the NDWI-derived images. 

To remedy this problem, Xu (2005) modified the NDWI (MNDWI) by using a middle-infrared band to substitute the near-infrared band [23]. The MNDWI is expressed as follows:

(9) MNDWI=(float(b1)−float(b2))/(float(b1)+float(b2))

where *b*1 is the green band and *b*2 is the mid-infrared band.

River System Pattern Evaluation Method

Under the influence of urbanization, intuitive changes take place in river networks. A quantitative description of the changes in the water system in a study area can improve the exploration of the mechanism and cause of change [24]. According to the regional characteristics and morphological indicators of the region, the river length and water surface area of different periods were selected, and the structural characteristics were selected from the parameters, such as river network density, river network water surface rate, connection rate, and connectivity, to analyze the evolution of the water system pattern in the study area (Table 2). 

The main characteristics were calculated as follows:

(10) $$D_{R}=L_{R}/A$$

(11) $$W_{P}=\frac{A_{\omega }}{A}\times 100\%$$

where $D_{R}$ is the density of the river network, representing the degree of development of the river length; $L_{R}$ and A are the total length of the river network and the total area of the study area; $W_{P}$ is the water surface rate, represents the degree of change in river area; and $A_{\omega }$ is the total area of rivers within the region.

The index selected in this system is the connectivity index in landscape ecology [25]. In river networks, the river course is defined as a line, the river junction is defined as a node, and the river course connecting two nodes is defined as a river chain [26]. Presently, the line-point rate β and network connectivity degree of the water network γ is mostly used to measure and evaluate the topology, connectivity, and complexity of the network. The calculation formulas for each index are as follows:

(12) β=L/V, 0≤β<3, 3≤V

(13) $$\gamma =L/L_{\max}=L/3(V-2),\;0\leq \gamma \leq 1$$

where L is the number of river chains in the network diagram; V is the number of nodes; $L_{\max}$ is the maximum possible number of river chains; and β is the ratio of the number of river chains to the number of nodes. When β is less than 1, the water system is a tree structure; and when β is greater than 1, the water system is a lattice structure or loop structure. γ is the ratio of the actual number of river chains to the maximum possible number of river chains, reflecting the actual connectivity between river channels. When the value is between 0 and 1 and close to 1/3, the river system is a tree-like structure. When it is close to 1, all nodes of the water system are connected. 

#### 2.2.3. Land-Use Change Matrix

The land-use change matrix method was used to study the change of the water area and other land types during the urbanization process. The land-use change matrix method is described in the references [27,28]. The reference formula is as follows:

(14) $$D_{\mathrm{ij}}=\left[\begin{array}{cccc} d_{11} & d_{12} & \cdot \cdot \cdot & d_{1n}\\ d_{21} & d_{22} & \cdot \cdot \cdot & d_{2n}\\ \vdots & \vdots & \ddots & \vdots \\ d_{n1} & d_{n2} & \cdot \cdot \cdot & d_{nn} \end{array}\right]$$

where $d_{ij}$ represents the area; n represents the number of land-use types; and i,j represents the coverage types at the beginning and end of the study period, respectively.

## 3. Result

### 3.1. The Urbanization Level of Zhengzhou

Using the entropy value calculation steps, 288 original data points of 10 indicators in study area from 1992 to 2015 were processed, and the corresponding weight values ($g_{j}$, the coefficient of difference) were calculated as shown in Table 3. 

As shown in the calculation of the weighted results, among the selected indicators of the comprehensive evaluation system of urbanization level, the weight ratio of GDP, population density, and urban built-up area rank in the top three, while the weight of tertiary industry accounts for the weakest proportion of GDP. Furthermore, economic growth, population, and land urbanization are the main factors in the urbanization process of Zhengzhou.

**Figure 2:**
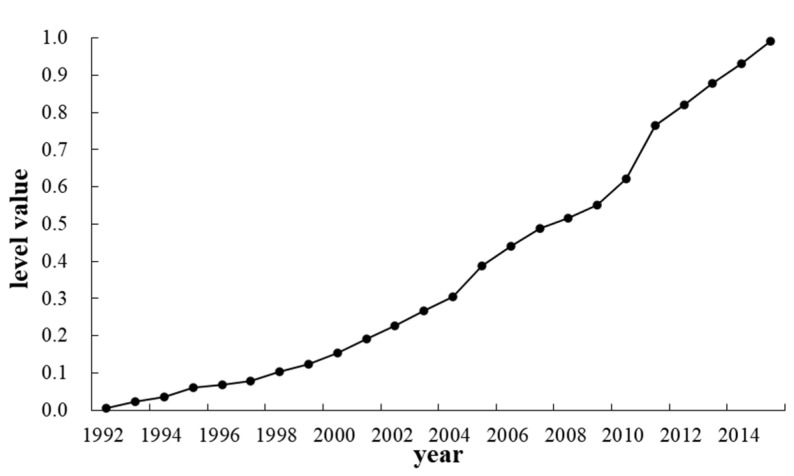
Level of urban development.

A quantitative evaluation of the development level of the urbanization process in Zhengzhou based on an analysis of the selected index data is shown in Figure 2.

The development process shows that the level of urbanization in Zhengzhou city continued to increase from 1992 to 2015 and was presented as three stages: (1) the stage of contiguous development from 1992 to 2002. The 14th National Congress in 1992 established the goal of developing a socialist market economy. Zhengzhou has nearly doubled in size and is in a phase of rapid development; (2) the period from 2003 to 2007. During this period, China’s accession to the World Trade Organization ushered in new opportunities and challenges in economic development. To adapt to the trend of economic globalization and create new economic growth points in the new century, plans were made to construct the Zhengdong New District, expand the city scale, and complete the leapfrog development; (3) from 2008 to 2015, Zhengzhou intensified its urbanization construction, optimized its industrial structure, and promoted the transformation and upgrading of traditional industries to achieve coordinated economic development and bring Zhengzhou’s urbanization to a new level. The changes in urban population and urban area in Zhengzhou from 1992 to 2015 are shown in Table 4.

### 3.2. Analysis of the Evolution of Zhengzhou’s Water System

**Figure 3:**
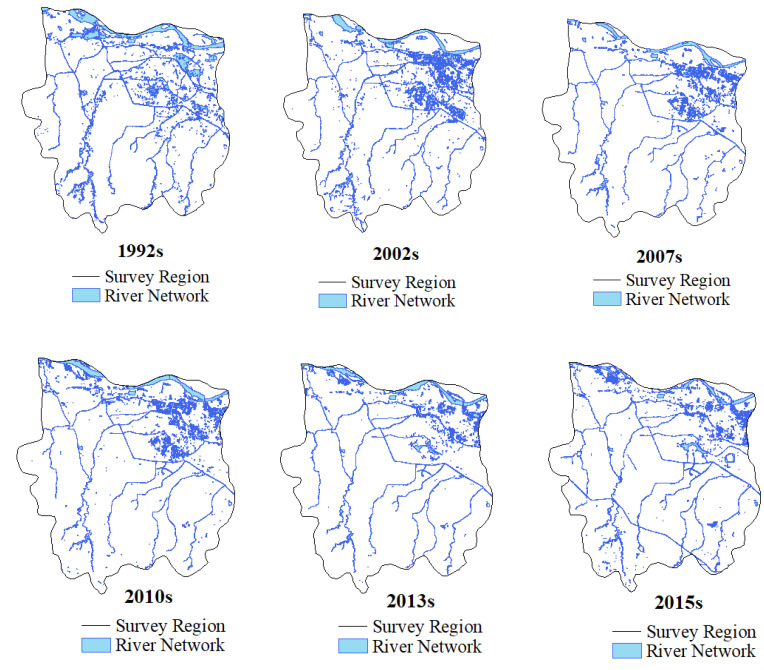
Schematic diagram of river network evolution in Zhengzhou in various periods.

**Figure 4:**
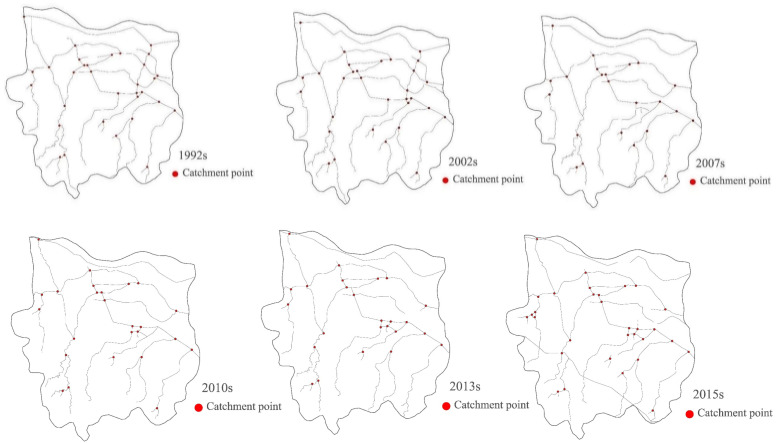
Schematic diagram of the evolution of the water system in Zhengzhou in various periods.

Artificial neural network classification and MNDWI technology were used to extract information about water bodies in the study area in different periods to determine the pattern of dynamic water system evolution (shown in Figure 3 and Figure 4).

Based on the qualitative analysis of the dynamic evolution of the water system in Zhengzhou, it was found that the water area experienced an obvious shrinking trend over the past 34 years. The river in the southwest area of Zhengzhou is significantly degraded, and the completion of the middle route of the South-to-North Water Transfer Project and the beginning of water supply are the main reasons for the increase in the river network density seen in 2015.

By studying the changes in river water grids during different periods, we can provide statistics such as river length *L_R_*, water area *Aω*, river network density *D_R_*, water surface rate *W_P_* (%), water system connectivity *γ*, and node connection rate *β*. Between 1992 and 2002: the river length decreased by an average of 3.83 km per year; the water area decreased by 14.47 km^2^; the river network density decreased from 0.47 to 0.43; the water surface ratio and nodal connectivity decreased by 0.014 and 0.06, respectively; the water system connectivity decreased from 0.46 to 0.44; and in terms of the rate of decrease and decline, the overall damage to the water system between 2002 and 2007 was greater than that between 1992 and 2002. During the 2007 to 2010 period: the overall water system pattern index value increased and the structure of the water system improved; the river length increased from 322.17 km to 415.30 km; the water area increased from 50.91 km^2^ to 55.07 km^2^; the river network density increased to 0.39; the water surface ratio increased from 0.048% to 0.052%; nodal connectivity increased by 0.09; and water system connectivity increased from 0.39 to 0.42. Between 2010 and 2013, there was a slight deterioration, and some of the water system pattern indicators recovered to the 2010 level between 2013 and 2015.Detailed data of the water pattern index are shown in Table 5. Research and analysis showed that the quantity index, structural characteristic index, and connectivity index of the river network in Zhengzhou city decreased and then increased, which indicated that the structure of Zhengzhou’s water system tended to first deteriorate, and then improve. These changes may be related to the implementation of river regulations and the introduction of artificial wetlands in Zhengzhou in 2007.

### 3.3. Analysis of the River Network Change Matrix 

In 1992, the area of cultivated land, forest, and grassland occupied the majority of all types of land use in Zhengzhou, accounting for 78.68% of the total area of the city, among which the area of cultivated land reached 535.33 km^2^, accounting for 50.40% of the total area of the city, and the area of water accounted for 7.86%. In 2002, the area of cultivated land remained in first place, while the area of construction land surpassed the area of forest and grassland, reaching second place. In 2007, the area of construction land exceeded the area of arable land and rose to first place, accounting for 422.56 km^2^, or 39.77%, of the total area. In 2010, the proportion of construction land increased to 43.64%, and the area of water was 55.07 km^2^, accounting for 5.18%. In 2013, the proportion of construction land was 51.35%, while the proportion of cultivated land, forest, and grassland shrank to 44.28%. The proportion of construction land reached 58.50% in 2015. At this time, the grassland and arable land area was 247.76 km^2^ and 144.19 km^2^, respectively, accounting for 36.89%. 

**Figure 5:**
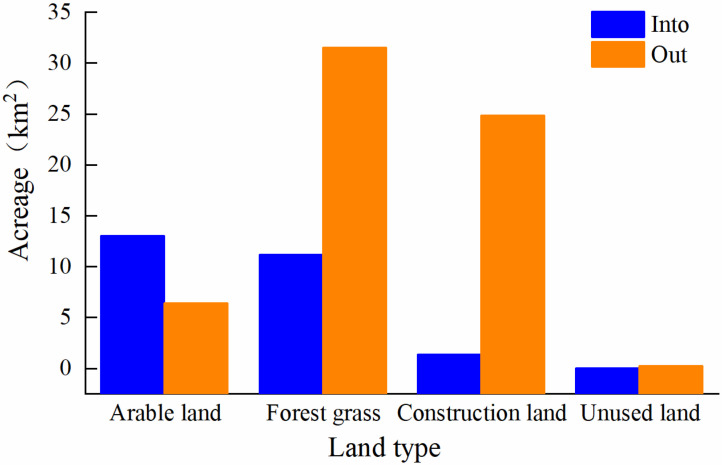
Analysis of water and other land types. Note: “Into” represents the transformation of other land-use types into water areas, and “Out” indicates the transformation of water areas into other land-use types.

The process of change in land-use types in Zhengzhou from 1992 to 2015 was analyzed (Table 6). The results show that the process of change from water areas to other land-use types is clear. The proportion of arable land, forest, grassland, and construction land that was transferred to water areas is less than 4%. Further analysis of the transfer-in and transfer-out relationship between water areas and other land-use types (Figure 5) shows that from 1992 to 2015, a total of 25.56 km^2^ of different types of land was transferred into water areas, among which arable land was the main contributor, accounting for 50.94% of the total transfer-in area. The amount of water area turned into forest and grassland, construction land, arable land, and unused land was 31.56 km^2^, 24.86 km^2^, 6.41 km^2^, and 0.26 km^2^, respectively, with a total area of 63.11 km^2^. To summarize, the transfer-out area is larger than the transfer-in area, which has destroyed the natural morphological structure of the river waters to a certain extent. 

### 3.4. Correlation Analysis of Water System Changes and Land-Use Types 

**Figure 6:**
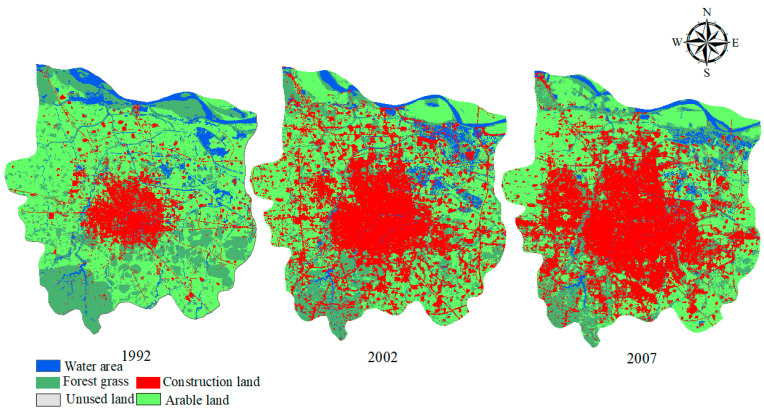

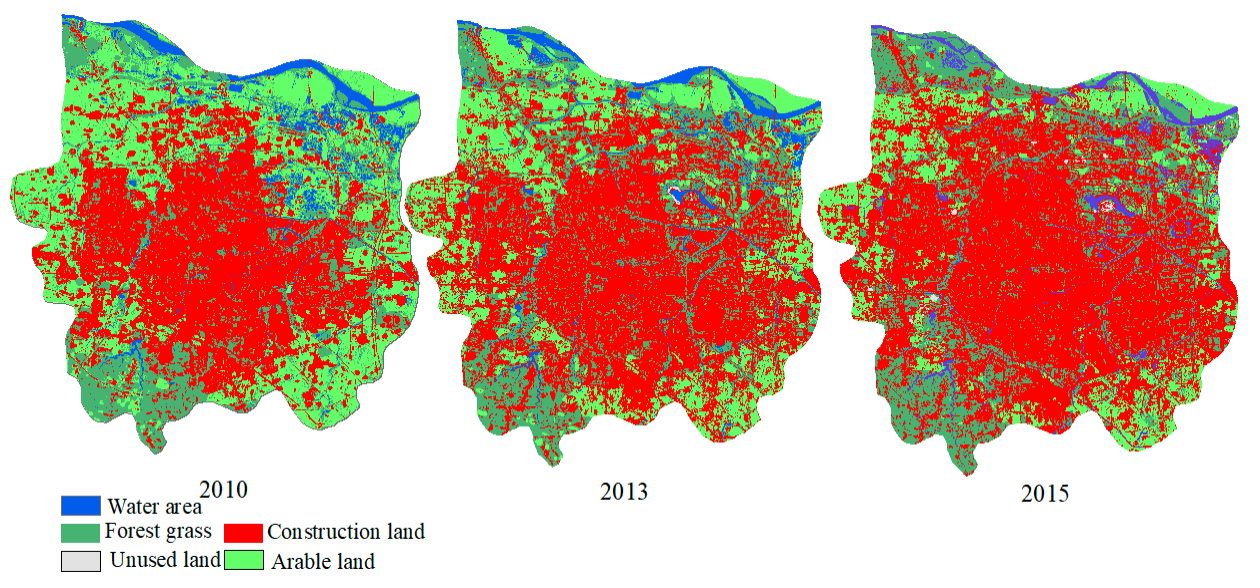
Land-use classification results from the 1992 to 2015 period.

The remote sensing interpretation images of Zhengzhou from different periods are shown in Figure 6. SPSS software (International Business Machines Corporation, New York, USA) was used to test the KS (Kolmogorov-Smirnov) normal distribution of the morphological structure indicators, such as the river network system, land-use type, and urbanization development level index. According to the results of the normal distribution test, a correlation analysis was performed on the morphology of the river network system, the structural characteristics index, and the proportion of each land-use type area, and a Pearson correlation analysis was performed on the data that satisfied the normal distribution test.

The normal distribution test results show that all the data regarding the structural characteristics of the river network conform to the vertical normal distribution. Among these, the types of land use and the proportion of construction land area, unused land, forest grassland, cultivated land, and water area all met the normal distribution test. A correlation analysis was then conducted between the five types of land and the structural indicators of each river network, with the results shown in Table 7.

The results show that the special indicators of the river network structure ($L_{R}$, Aω, $D_{R}$, $W_{P}$, β and γ) are significantly correlated with construction land, and their correlation coefficients are all below 0.05. Aω and $W_{P}$ are significantly correlated with water area and cultivated land; the correlation coefficient with water area is at the 0.01 level, and the correlation coefficient with cultivated land is at the 0.05 level. Although there are certain correlations between the indicators of the river network structure characteristics and other types of land use, the correlation between the two is not significant. This shows that there is a certain internal connection between the river network structure and land-use change; however, the construction land proportion is closely related to the river network structure characteristic index, which indicates that construction land is the main driving factor for the change in the water system pattern. It should also be noted that construction land is negatively correlated with the water pattern of Zhengzhou, and unused land is only negatively correlated with Aω and $W_{P}$.

## 4. Discussion

### 4.1. Relationship between Urbanization Level and River Network Water System Response

Various influencing factors cause changes in river network structures in urban areas, including geology, topography, climate, and human activities. In the short term, human activities are the main influencing factor for changes in the river system [29]. According to the river network evolution diagram (Figure 3) and the land-use classification map (Figure 6) for Zhengzhou covering different periods, the reason for the reduction in the number of tributaries and the simplification of the structure of the river network in Zhengzhou from 1992 to 2015 was that the expansion of construction land encroached on the tributary channels. From the number of changes in the water regime pattern indicators and land-use transfer matrix in Zhengzhou city from 1992 to 2015, it is clear that the shrinkage of the river network and the extensive hardening of the land surface in Zhengzhou city region, leading to changes in the underlying surface runoff, is likely to affect the hydrological cycle processes in the area. Therefore, it is necessary to understand the level of urbanization in the study area and its level of influence of the river network.

The regression analysis method was used to quantitatively describe the relationship between the structural characteristics of the water system and the urbanization level, and a curve regression equation representing the urbanization level and the structural characteristics and connectivity of the river network was obtained (Table 8), with the value of *x* indicating the urbanization level index.

### 4.2. Drivers of Structural Change in Water Systems

As urbanization develops, humans are hardening the land surface with pavements and channels to beautify the urban living environment. When precipitation occurs, the runoff and confluence time period is greatly shortened, resulting in a larger peak flow [30]. Simultaneously, to meet the requirements of irrigation and water supply, a large number of water conservancy projects have been undertaken, which have changed the shape of lakes and their natural hydrological processes, having a major impact on the pattern and connectivity of river networks and the hydrological situation of rivers [31]. In addition, due to unsteady channel shapes in different sections, the flow of natural channels is often affected by sidewall turbulence [32]. During a flood, suspended sediment transport usually ranges from low to high concentration, depending on the local flow and ground conditions [33].

In the past 34 years, urbanization has developed rapidly in Zhengzhou. Although Luo et al. (2021) studied the influence of the trend of urbanization on the river network structure in Zhengzhou and obtained similar results to this article, unlike this paper, they believe that the facilities, economy, and population of the city have equal weight values on urbanization [34], which we believe is unreasonable. We further provide an evaluation of the inner relationship between the urbanization level and the characteristic values of the river network structure. In 2007, the comprehensive score of urbanization was 0.48, and each index of the water system pattern had reached the worst value. Since then, Zhengzhou has started constructing an ecological water system from a single river treatment to a comprehensive treatment of the rivers and lakes. In 2010, Zhengzhou’s ecological water system project was put into operation, with the first phase of treatment of the Chaohe, Weihe, and Suoxu rivers, the Yellow River diversion project, and the source replenishment of the Dongfeng canal basically completed, realizing the goal of water access. By 2015, the comprehensive urbanization score was 0.97, the river length had recovered from 322.17 km in 2007 to 449.52 km, the connectivity of the river system had increased from 0.39 to 0.43, and the river network was greatly improved. Considering that construction land is the main driving factor of the change in the water system pattern of the northern plain, the influence of construction land on the local water system pattern should be fully appreciated in the future.

## 5. Conclusions

1. From 1992 to 2015, the conversion of land-use types in Zhengzhou was mainly the transfer of cultivated land, forest and grassland, and water areas to construction land. Among them, construction land increased from 13.43% to 58.50%, and the proportion of cultivated land dropped by 36.83%. The water, forest, and grassland areas showed a decreasing trend in different periods.
2. The MNDWI method was used to extract the river network map, and an analysis of the various structural characteristics indicators of the river morphology and connectivity in six different periods from 1992 to 2015 showed that the river length *L_R_* decreased from 496.22 km in 1992 to 322.17 km in 2007, and recovered to 449.52 km in 2015. The water area *Aω* decreased from 83.51 km^2^ to 50.91 km^2^ in 2007, and was steady at approximately 45.94 km^2^ by 2015. The nodal connectivity *β* decreased from 1.32 in 1992 to 1.08 in 2007, and recovered to 1.19 in 2015. The water system connectivity *γ* decreased from 0.46 in 1992 to 0.39 in 2007, increasing to 0.43 in 2015.
3. The transferred water area destroyed the natural form of the river. There is a significant correlation between the river network structure and the characteristic indicators of construction land, with the correlation coefficients all below 0.05. However, the proportion of built-up land is closely related to the structural characteristics of the river network, which indicates that built-up land is the main driving factor for changes in the water system pattern.
4. Regression analysis quantitatively described the relationship between the level of urbanization and the structural characteristics of the river system. The level of urbanization is significantly related to river length, water area, river network density, water surface velocity, connection rate, and connectivity. For the next step, in the planning of urban spaces in Zhengzhou, a landscape pattern in which rivers and green spaces are connected in a series should be created. While promoting the ecological protection of the river with the help of the existing forest land and water surface, the stability of the river network structure can be maintained and assistance for the development of the length of the river can be provided.

## Figures and Tables

**Table 1:** Evaluation index system for measuring urbanization level.

Target Layer	Criterion Layer	Indicator Layer	Unit of Measure
Urbanization level	Population level	Total population of the city	Ten thousand
		Population density	Per km^2^
	Economic level	GDP	Ten thousand yuan
		Tertiary industry’s share of GDP	%
	Regional landscape	Built-up area	km^2^
		Green coverage rate in the built-up area	%
		Public area per capita	m^2^
	City facilities	Water supply pipe length	km
		Road length	km
		Road area	m^2^

**Table 2:** Index system of the connected forms of the water system.

Indicator Name	Unit	Calculation Method	Physical Meaning
Length of the river *L_R_*	km		The extent of regional water system development.
Water area *A_ω_*	km^2^		The extent of regional water system development.
River network density *D_R_*	km/km^2^	$D_{R}=L_{R}/A$	The length of the river in a unit area and the development of the length of the river.
Water surface coverage *W_P_*	%	$W_{P}=\frac{A_{\omega }}{A}\times 100\%$	The ratio of the area of the river to the whole area and the development of the area of the river.
Node connection rate *β*	Dimensionless	β=L/V	The ratio of the number of river chains to the number of nodes in the water system network reflects the average number of chains connected to each node.
Water system connectivity *γ*	Dimensionless	$\gamma =L/L_{\max}=L/3(V-2)$	The actual degree of connection between nodes.

**Table 3:** Empowerment of the comprehensive measurement evaluation index of urbanization level (1992–2015).

Target Layer	Criterion Layer	Indicator Layer	Unit of Measure	Weight Value
Urbanization level	Population level	Total population of the city	Ten thousand	0.0960
		Population density	Per km^2^	0.1521
	Economic level	GDP	Ten thousand yuan	0.1642
		Tertiary industry’s share of GDP	%	0.0264
	Regional landscape	Built-up area	km^2^	0.1212
		Green coverage rate in built-up area	%	0.0672
		Public area per capita	m^2^	0.1055
	City facilities	Water supply pipe length	km	0.0907
		Road length	km	0.0713
		Road area	m^2^	0.1053

**Table 4:** Changes in urban population and urban area in Zhengzhou.

Years	Urban Population (Million)	Percentage Increase (%)	Urban Area (km^2^)	Percentage Increase (%)
In 1992	1.869	–	93.10	–
1992–2002	2.783	48.90	156.40	67.99
2003–2007	3.189	14.59	302.00	93.30
2008–2015	4.893	53.43	437.60	44.90

**Table 5:** Index data of the water system pattern in Zhengzhou.

	Years	1992	2002	2007	2010	2013	2015
Indicators							
River length *L_R_* (km)		496.22	454.10	322.17	415.30	408.67	449.52
Water area *A_ω_* (km^2^)		83.51	69.04	50.91	55.07	45.73	45.94
River network density *D_R_* (km/km^2^)		0.47	0.43	0.30	0.39	0.38	0.42
Water rate *W_P_* (%)		0.079	0.065	0.048	0.052	0.043	0.043
Node connection rate *β*		1.32	1.26	1.08	1.17	1.14	1.19
Water system connectivity *γ*		0.46	0.44	0.39	0.42	0.41	0.43

**Table 6:** The conversion area proportion of different land-use types (unit: %).

Land Type	Arable Land	Forest Grass	Water Area	Construction Land	Unused Land
Arable land	19.81	18.37	2.43	59.10	0.28
Forest grass	9.92	35.05	3.72	51.05	0.26
Water area	7.69	37.83	24.35	29.83	0.31
Construction land	1.20	8.56	0.95	88.93	0.35
Unused land	12.30	23.66	3.51	58.76	1.78

Note: The rows are the weight of area transferred out and the columns are the weight of area transferred in.

**Table 7:** Characteristic indexes of water system pattern and Pearson analysis of proportion of each land use.

Indicators		Waters	Grassland	Arable Land	Construction Land	Unused Land
$L_{R}$	Pearson correlation	0.631	0.676	0.278	0.901 *	0.145
	Significance (bilateral)	0.179	0.141	0.594	0.024	0.784
Aω	Pearson correlation	1.000 **	0.769	0.911 *	0.836 *	0.518
	Significance (bilateral)	0.000	0.074	0.012	0.045	0.292
$D_{R}$	Pearson correlation	0.660	0.682	0.311	0.890 *	0.116
	Significance (bilateral)	0.154	0.136	0.549	0.040	0.827
$W_{P}$	Pearson correlation	1.000 **	0.770	0.914 *	0.833 *	0.524
	Significance (bilateral)	0.000	0.073	0.011	0.047	0.286
β	Pearson correlation	0.663	0.689	0.309	0.806 *	0.248
	Significance (bilateral)	0.151	0.130	0.552	0.048	0.636
γ	Pearson correlation	0.604	0.677	0.240	0.889 *	0.0313
	Significance (bilateral)	0.204	0.140	0.648	0.042	0.546

* Significant correlation at the 0.05 level (bilateral); ** significant correlation at the 0.01 level (bilateral).

**Table 8:** Correlation between urbanization level and characteristic values of the river network water system structure.

Dependent Variable (*y*)	Curve Regression Equation	*R* ^2^
$L_{R}$	*y* = 520.388 − 517.539*x* + 455.179*x*^2^	0.677
Aω	*y* = 85.925 − 87.118*x* + 47.648*x*^2^	0.966
$D_{R}$	*y* = 0.494 − 0.501*x* + 0.435*x*^2^	0.676
$W_{P}$	*y* = 0.081 − 0.082*x* + 0.045*x*^2^	0.966
β	*y* = 1.365 − 0.825*x* + 0.712*x*^2^	0.739
γ	*y* = 0.472 − 0.239*x* + 0.212*x*^2^	0.718

## Data Availability

Not applicable.
